# Continuous Optical Biosensing of IL-8 Cancer Biomarker Using a Multimodal Platform

**DOI:** 10.3390/bioengineering12101115

**Published:** 2025-10-17

**Authors:** A. L. Hernandez, K. Mandal, B. Santamaria, S. Quintero, M. R. Dokmeci, V. Jucaud, M. Holgado

**Affiliations:** 1 Group of Optics, Photonics, and Biophotonics, Center for Biomedical Technology (CTB), Universidad Politécnica de Madrid, Parque Científico y Tecnológico de la UPM, Campus de Montegancedo, Pozuelo de Alarcón, 28223 Madrid, Spain; beatriz.santamaria@ctb.upm.es (B.S.); sergio.quintero@inl.int (S.Q.); 2 Terasaki Institute for Biomedical Innovation, 21100 Erwin St, Woodland Hills, CA 91367, USA; kalpana.mandal@gmail.com (K.M.); mdokmeci@terasaki.org (M.R.D.); vjucaud@terasaki.org (V.J.); 3 Group of Organ and Tissue on-a-chip and In-Vitro Detection, Health Research Institute of the Hospital Clínico San Carlos, IdISSC, C/Profesor Martín Lagos s/n, 4ª Planta Sur, 28040 Madrid, Spain; 4 Department of Mechanics, Chemistry and Industrial Design Engineering, Escuela Superior de Ingeniería y Diseño Industrial, Universidad Politécnica de Madrid, Ronda de Valencia 3, 28012 Madrid, Spain; 5 Medical Devices Group, International Iberian Nanotechnology Laboratory, Av. Mestre José Veiga s/n, 4715-330 Braga, Portugal; 6 Department of Applied Physics and Materials Engineering, Escuela Técnica Superior de Ingenieros Industriales, Universidad Politécnica de Madrid, C/José Gutiérrez Abascal, 2, 28006 Madrid, Spain

**Keywords:** continuous sensing, fluidic device, integrated module, fiber optic sensors, label-free, microphysiological systems, optical biosensors

## Abstract

In this work, we used a label-free biosensor that provides optical readouts to perform continuous detection of human interleukin 8 (IL-8), which is especially overexpressed in certain cancers and, thus, could be an effective biomarker for cancer prognosis estimation and therapy evaluation. For this purpose, we engineered a compact, portable, and easy-to-assemble biosensing module device. It combines a fluidic chip for reagent flow, a biosensing chip for signal transduction, and an optical readout head based on fiber optics in a single module. The biosensing chip is based on independent arrays of resonant nanopillar transducer (RNP) networks. We integrated the biosensing chip with the RNPs facing down in a simple and rapidly fabricated polydimethyl siloxane (PDMS) microfluidic chip, with inlet and outlet channels for the sample flowing through the RNPs. The RNPs were vertically oriented from the backside through an optical fiber mounted on a holder head fabricated ad hoc on polytetrafluoroethylene (PTFE). The optical fiber was connected to a visible spectrometer for optical response analysis and consecutive biomolecule detection. We obtained a sensogram showing anti-IL-8 immobilization and the specific recognition of IL-8. This unique portable and easy-to-handle module can be used for biomolecule detection within minutes and is particularly suitable for in-line sensing of physiological and biomimetic organ-on-a-chip systems. Cancer biomarkers’ continuous monitoring arises as an efficient and non-invasive alternative to classical tools (imaging, immunohistology) for determining clinical prognostic factors and therapeutic responses to anticancer drugs. In addition, the multiplexed layout of the optical transducers and the simplicity of the monolithic sensing module yield potential high-throughput screening of a combination of different biomarkers, which, together with other medical exams (such as imaging and/or patient history), could become a cutting-edge technology for further and more accurate diagnosis and prediction of cancer and similar diseases.

## 1. Introduction

The tumor-promoting role of IL-8 has been demonstrated in different types of cancer, including melanoma, prostate, colon, pancreatic, breast, and lung [1]. IL-8 is a member of the CXC chemokine family secreted by stromal cells (endothelial cells and fibroblasts) [2]. IL-8 signaling activates multiple upstream signaling pathways, such as angiogenic responses, cancer cell migration, proliferation, and survival [3]. IL-8 has been widely studied and shown great promise as an early diagnostic and predictive cancer biomarker [4]. Its detection as a cancer biomarker can be applied as an alternative approach to better understand cancer diseases at the molecular level and improve disease monitoring and modeling.

Traditionally, the enzyme-linked immunosorbent assay (ELISA) has been the gold standard for biomarker detection [5]. However, it presents several constraints: it is a laborious procedure, requires a large sample volume, has limited options for multiplexing, and requires chemical development or labels for the signal output measurement [6] which does not allow continuous measurement. This last feature prevents their application to point-of-care (PoC) technologies, which are intended to be portable devices that can provide reliable test results rapidly, simply, and cost-effectively [7]. The use of label-free biosensors as an alternative to biomarker detection in vitro has been widely studied [8] (such as SPR and EC). They have the capacity to use small sample volumes and detect low-molecular-weight molecules, and they are suitable for implementation in lab-on-a-chip devices and PoC systems [9]. More specifically, among the different biosensing transducers, according to the detected transducing signal nature used (electrochemical, optical, piezoelectric, mechanical, or magnetic) [10], optical label-free biosensors seem to hold significant competitive advantages relative to other approaches [11,12,13]. One of these advantages is their suitability for miniaturization and integration into devices such as PoC.

In addition, the capacity to continuously monitor specific molecules from the biological systems is a key factor for improving and evolving PoC detection systems based on label-free biosensing to increase their functionality in medical applications [14], specifically for drug screening, disease monitoring, disease modeling, and fundamental research. In contrast to endpoint biosensors, continuous biosensors can provide relevant information during a specific timeframe where dynamic biological processes occur [15,16,17]. Continuous biosensors are a promising tool for studying biodynamic systems such as organ-on-a-chip technologies [18] and cancer microphysiological systems, where the tumor microenvironment needs to be further studied and better understood.

In this regard, optical label-free biosensors that can carry out continuous detection are an excellent option for performing ongoing measurements in which no secondary species or chemical developments are needed. Surface plasmon resonance (SPR) biosensors have traditionally been the mainstream technology for sensing biomolecules [19]. They have gained much attention in the last few decades for their sensitivity and ability to measure the kinetics and affinity of bimolecular complexes binding in real time. However, their operation mode, based on complex light coupling and expensive equipment, hinders their adequacy for in-line sensing of organ-on-a-chip technologies and in vivo continuous sensing.

We previously demonstrated the ability of optical RNP transducers to measure refractive index (RI) variations in real time in a high-throughput multiplexed format [20]. Also, we showed the capacity of RNPs to detect biological species [21]. Here, we developed a microfluidic device for continuous detection of IL-8, a cancer biomarker, by using a stand-alone integrated optical sensing module based on RNPs to be used in multiple applications, such as in multi-modular platforms for in-line detection of cell-secreted biomarkers, from an organ-on-a-chip system. The LoD achieved was 22.7 ng/mL, which is within the range of cell-secreted IL-8 concentrations in tissue-like physiological systems, especially in cancer-ecosystems-on-a-chip models [22]. This new biosensing platform could allow for the generation of a continuous and uninterrupted stream of measurement data over a prolonged period, simply and cost-effectively, avoiding waiting hours or days to acquire data and analyze the results. Microfluidic devices for biomolecule detection are arousing the interest of the scientific community and stakeholders to be applied to disease monitoring approaches and learning about the biological processes occurring in certain pathologies, as well as the discovery and application of new drugs [23].

## 2. Materials and Methods

### 2.1. Sensing Principle: Optical Biosensor

The biological sensor developed in this work is a label-free biosensor composed of an optical transducer based on RNPs (sensing chips) and an optical fiber bundle for light emission and signal detection. RNPs are composed of 10 pairs of Bragg reflectors of silicon nitride and silicon oxide (Si_3_N_4_/SiO_2_) and a central cavity of silicon oxide (SiO_2_) arranged on a quartz substrate [21]. A resonant nanopillar has an optical response consisting of a spectral band gap that prevents light transmission (photonic gap), except in a specific range of the band, where the light is transmitted (resonance) due to the central cavity-forming resonant mode. The light is guided through each RNP. Due to their nanometric size and close arrangement, a portion of the light extends beyond the surface of the nanopillars into the surrounding medium. This phenomenon, known as evanescent field detection, allows light to interact with and sense molecules or materials present on the surface of the nanopillars.

**Figure 1:**
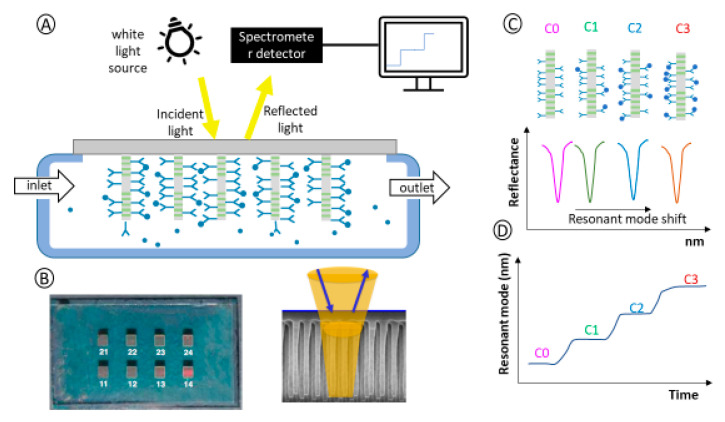
(**A**) Continuous flow measurement scheme and optical interrogation. (**B**) Photo of the sensing chip with RNP arrays for potential multiplexed detection (**left**). SEM photo of RNPs and light confinement among them (**right**). (**C**) RNP detection of increasing concentrations of biomarkers (C0, C1, C2, C3) associated with the optical resonant mode shift along the wavelength upon vertical interrogation of the RNPs. (**D**) Subsequent sensogram representation (time vs. resonant mode shift).

For biosensing, RNPs are integrated into a fluidic chip with their backside exposed, enabling the biological sample to flow through and infiltrate the matrix formed by the RNPs. Optical interrogation is performed from this backside, allowing for precise monitoring of several key events: changes in the optical properties of the liquid, the immobilization of a bioreceptor on the RNP surface, and the specific affinity interactions between the bioreceptor and the target molecule (analyte) being detected. This setup facilitates continuous observation of these interactions for effective biosensing (Figure 1A). RNPs are grouped in cells or arrays arranged in different zones of the same chip that, once immobilized with the bioreceptor, are called BICELLs (Bio Photonic Sensing Cells). This distribution allows RNPs to be used as sensors with a high multiplexing capacity to detect different biomolecules in the same chip (Figure 1B). When vertically interrogated, RNPs produce a very narrow resonant mode that shifts in wavelength (nm) as a function of the biological material (concentration) immobilized on their surface (Figure 1C). In continuous biosensing, the resonant mode can be monitored over time, and its shift can be observed as different biomolecule concentrations flow through the RNPs (Figure 1D).

### 2.2. Biosensing Measurements

A label-free immunoassay in a direct format was developed to continuously detect IL-8 using the platform described in this study. Initially, anti-IL-8 antibodies were immobilized on the surface of the RNPs, followed by a blocking step with BSA to ensure specific recognition and detection of IL-8. This approach required no additional chemical modifications or labeling procedures. The reusability of the biosensing system was evaluated by regenerating the RNP sensing surface to its anti-IL-8 functionalized state through manual injection of 1 nM HCl for 5 s using a syringe. This study utilized a single high concentration of IL-8 to demonstrate the platform’s capabilities. Future work will involve optimization for specific applications, including the establishment of concentration gradients to define linear response ranges and quantitative performance. Such optimization will be essential for adapting the platform to real-world diagnostic scenarios.

#### 2.2.1. Chemical Surface Modification of RNPs

For the biofunctionalization (receptor immobilization) of the RNPs, a surface chemical modification protocol was applied specifically for antibody immobilization. The process is based on silane chemistry, using (3-ethoxydimethylsilyl)propylamine, APDMS, and carbonyl diimidazole (CDI) linkers for functional amine activation. Afterward, the antibody is immobilized by reacting it with the activated amine and COOH groups in the constant region of the antibody. We followed the protocol from our previous publication [23,24].

#### 2.2.2. Reagents Used for the Continuous Biosensing Assay

Phosphate-buffered saline (PBS), pH 7.4, came from Sigma Aldrich, St. Louis, Missouri, USA, ref P3813. Hydrochloric acid (HCl), 1 M, came from Sigma Aldrich, St. Louis, Missouri, USA, ref 1.10165. APDMS (18306-79-1 Sigma Aldrich, St. Louis, Missouri, USA). CDI (115533 Sigma Aldrich, St. Louis, Missouri, USA). Purified anti-human IL-8 monoclonal mouse antibody came from Biolegend, San Diego, CA, USA, ref 511501. Recombinant Human IL-8 (carrier-free) came from Biolegend, San Diego, CA, USA, ref 574202.

## 3. Results and Discussion

### 3.1. Integrated Continuous Sensing Platform

#### 3.1.1. Design and Fabrication of the Fluidic Chip

**Figure 2:**
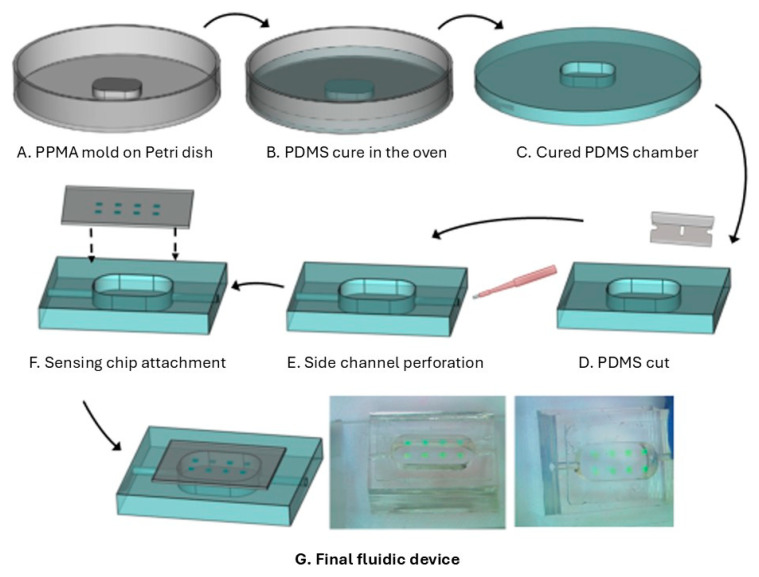
(**A**) Fabrication process of the fluidic chip used to host the sensing chip for continuous detection of IL-8. (**A**) PMMA mold glued to a Petri dish. (**B**) PDMS filling and curing. (**C**) PDMS peeled off. (**D**) Piece cut to hold the chip. (**E**) Side channels punched out for the tubbing connection. (**F**) The sensing chip is positioned with the RNPs facing downward, aligning precisely with the chamber area of the PDMS piece to facilitate direct contact between the RNPs and the liquid. The sensing chip is positioned with the RNPs facing downward, aligning precisely with the chamber area of the PDMS piece to facilitate direct contact between the RNPs and the liquid. (**G**) Final fluidic device. Photo on the right.

The fluidic chip was fabricated with PDMS (polydimethylsiloxane) (Sylgard™ 184 Silicone Elastomer Kit) using soft lithography. For that, a PMMA (polymethyl methacrylate) mold of 14 × 6 × 1.5 mm dimensions was cut (to form a chamber of 126 uL) using laser-cutting equipment and attached to a plastic Petri dish for fluidic well casting. The Petri dish was filled with a mix of PDMS and elastomer, 10:1, and kept in a 70-degree oven for 3 h. After curing, the PDMS was peeled off and cut into a piece measuring 24 × 20 × 5 mm within the chamber (126 μL) in the center of the piece (Figure 2A–D). Afterward, two side channels were punched with a 1.5 mm biopsy punch, and 1 mm internal diameter tubing was connected at the outlet and inlet holes for the liquid flow. Then, the sensing chip was placed in direct contact with the fluidic chip to avoid leakage. RNPs fall into the chamber of the fluidic chip for contact with the reagents (Figure 2E–G).

#### 3.1.2. Fabrication and Design of the Optical Head for the Readout System Integration and Biosensing Module Clamping

**Figure 3:**
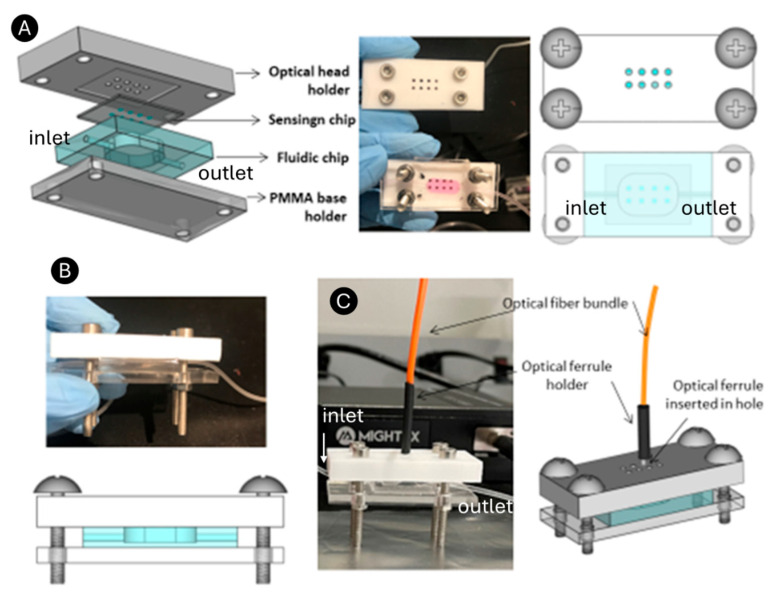
(**A**) Design of the optical head for multiplexing interrogation. (**B**) Clamping of the optical head and the fluidic chip together. (**C**) Optical bundle integration in the optical head.

To achieve the integrated optical interrogation of the RNPs, an ad hoc polytetrafluoroethylene (PTFE) optical head holder was designed and manufactured using a milling machine. The holder includes a sensing chip-shaped depression within eight circular holes, one for each RNP array (Figure 3A). The holes connect the backside of the RNPs to a bifurcated optical bundle (represented in orange) through an optical ferrule of the same diameter. The fluidic chip was positioned on a PMMA base with dimensions matching those of the optical head, and the two were securely clamped together using screws. This means that the sensing chip is mechanically coupled to the PDMS microfluidic chip so that the RNP array region is precisely aligned with the detection well of the fluidic chamber, which features lateral inlet and outlet channels. When both chips are properly aligned and sealed, fluid leakage is highly unlikely.

To ensure a tight and stable seal, the two chips are mounted within a custom PMMA holder consisting of a base supporting the microfluidic chip and a PTFE optical holder that also serves as the top cover, which applies uniform pressure to the sensing chip. The assembly forms a sandwich-like structure, secured by four screws positioned at the corners of the PMMA and PTFE parts. This setup creates a sandwich structure, with the fluidic chip and sensing chip positioned between the PMMA base and the optical head (Figure 3B). This configuration maintains constant pressure across the interface and ensures mechanical stability during fluid flow and optical measurements.

In this work, only one optical bundle was connected since we experienced the limitation of a single spectrometer for signal collection. However, this limitation can be remedied by using a beam splitter allocated at the spectrometer entrance for multiplexed signal acquisition, and thus, the biosensing module presented here (Figure 3C) could be used for multianalyte detection. Overall, we successfully developed a portable and easy-to-handle integrated sensing module.

Future studies will explore the integration of multiple spectrometers or beam splitters to demonstrate the feasibility of synchronous multi-biomarker detection, further expanding the platform’s applicability for complex diagnostic applications.

#### 3.1.3. Continuous Sensing Platform Integration

**Figure 4:**
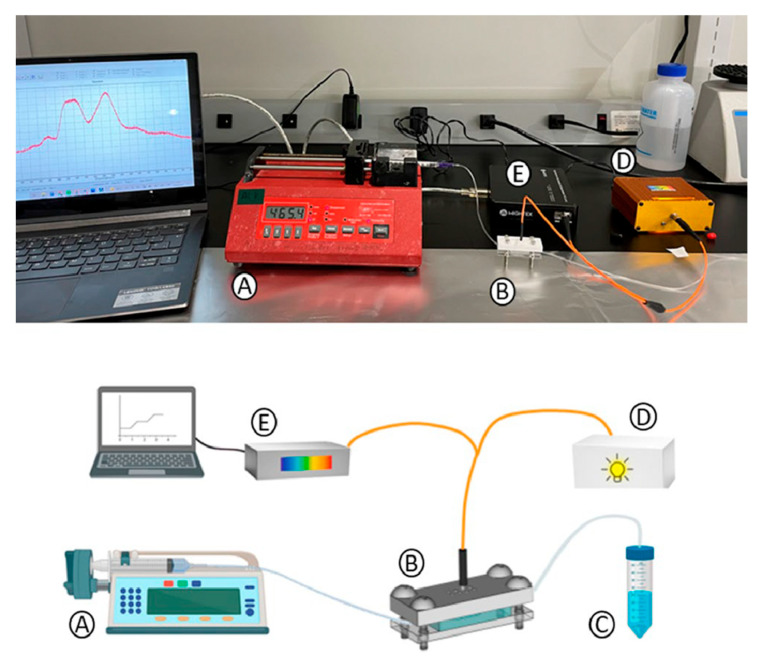
Continuous biosensing platform. A. Syringe pump, B. biosensing module, C. reservoir for waste accumulation, D. white light source, E. VIS-spectrometer connected to a computer for continuous observation.

As shown in Figure 4, a syringe pump (NE-1000 Programmable Single Syringe Pump), which flows the different reagents at 10 µL/min for different life spans required by the experiment, is connected to the biosensing module inlet. Different flow rates were tested to provide mechanical stability and sufficient interaction time between reagents for effective biosensor performance. The outlet tubing addressed the flowed reagents to a waste reservoir. For the optical interrogation, an optical bundle (manually fabricated ad hoc) bifurcated at one of the ends was used. The common end of the bundle is normally placed on the nanopillar array to stimulate RNPs and measure the reflected light. One fiber from the bundle, connected to a white light source (a miniature fiber optic VIS-NIR light source with a five-watt input and a Tungsten Krypton bulb), illuminates the nanostructures. The other fiber collects the reflected light from the transducers (RNPs) and directs it to a CCD spectrometer (Mightex HRS-VIS-005, high-resolution, high-stability CCD spectrometer). This setup generates an optical spectrum (intensity vs. wavelength), which can be monitored over time. There is a 150 s delay between the fluid pumping start point and the fluid filling of the chamber and later signal change observations. For every reagent that flowed, the pump was stopped, the syringe was manually changed, and pumping was resumed.

#### 3.1.4. Performance Evaluation of the Integrated Continuous Sensing Platform

The functionality of the biosensing module was rigorously tested to ensure proper fluidic operation and effective sensing performance. Specifically, the tests verified the absence of leakage and the precise control of liquid flow through the fluidic device. Additionally, the sensing module was evaluated to confirm optimal light alignment and the operational response of the resonant nanoparticle structures (RNPs) and the optical system. A bulk sensing experiment was conducted, studying the optical mode wavelength position as a function of different refractive indices (RIs) flowing through the system.

Discrete measurements were taken from each **BICELL** under dry conditions and when embedded in liquids of varying refractive indices (RIs), including water (RI = 1.33), ethanol (RI = 1.36), and hexane (RI = 1.37). The results demonstrated appropriate responses across all BICELLs on the sensing chip.

**Figure 5:**
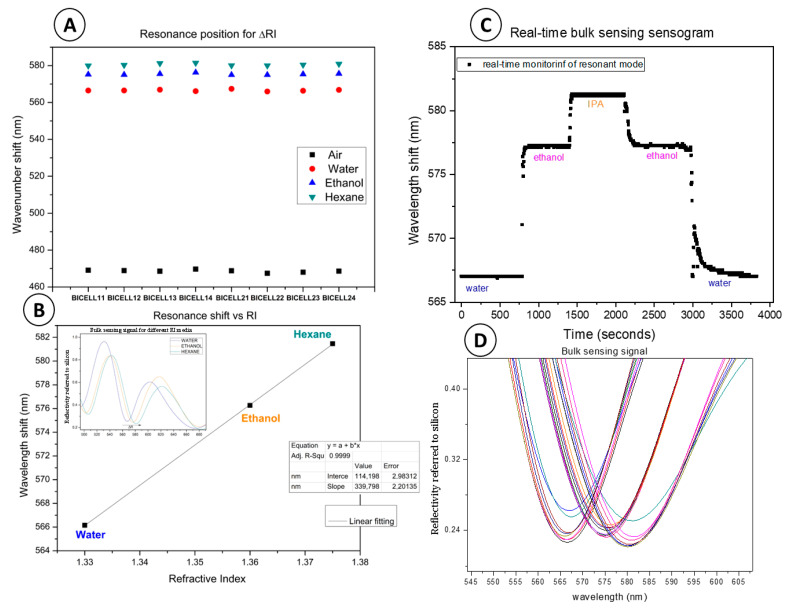
(**A**) Position in wavelength of the resonant mode of each BICELL exposed to the different RI liquids. (**B**) Resonant mode position as a function of the RIs of the fluids of one of the BICELLs. (**C**) Sensogram of one of the BICELLs showing resonant mode shift in time (under different RI fluids). (**D**) Resonant mode bulk signals of the RNPs for flowing water, ethanol, and IPA liquid.

Figure 5A shows the resonant mode wavelengths for the different RIs: approximately 470 nm in air, 566 nm in water, 578 nm in ethanol, and 580 nm in hexane.

Figure 5B illustrates the sensitivity calculation for one BICELL, derived from the resonance wavelength shift as a function of the RIs. The bulk sensitivity was determined as

Sensitivity = Δy/Δx = 14/0.37 = 350 nm/RIU/RIU

Subsequently, water, ethanol, and isopropanol (IPA) were flowed sequentially through the biosensing module for continuous detection. Water flowed first for 15 min at 100 μL/hour. Ethanol and IPA were then introduced sequentially, followed by additional cycles of ethanol and water.

This experiment, depicted in Figure 5C, generated a sensogram demonstrating the system’s capability to perform continuous measurements over an hour. The results confirmed the fluidic cell’s tightness, the RNPs’ stability, and the optical and systemic stability of the readout system. Figure 5D presents the resonant mode signals of the RNPs during the flow of water, ethanol, and IPA, illustrating the sensing module’s continuous response capabilities and robustness.

This comprehensive evaluation underscores the biosensing platform’s reliability and readiness for subsequent biological detection protocols.

Custom software developed in MATLAB 2024B facilitated continuous measurements and data acquisition for this experimental setup.

The integrated continuous sensing platform was designed to enable real-time, label-free detection of biomarkers using resonant nanopillars (RNPs) as the core sensing element. The platform consists of three main components: the fluidic chip, the optical head, and the readout system, each meticulously engineered to ensure seamless integration and high-performance operation.

The modular design of the platform allows for scalability and adaptability. Future work will focus on incorporating multiple spectrometers or beam splitters to enable synchronous multi-biomarker detection, further enhancing the platform’s applicability in complex diagnostic scenarios. Additionally, optimization of fluidic and optical components will be explored to improve sensitivity, reduce response time, and expand the range of detectable analytes.

### 3.2. Sensing Signal Processing in Biological Detection Using the Platform Described

**Figure 6:**
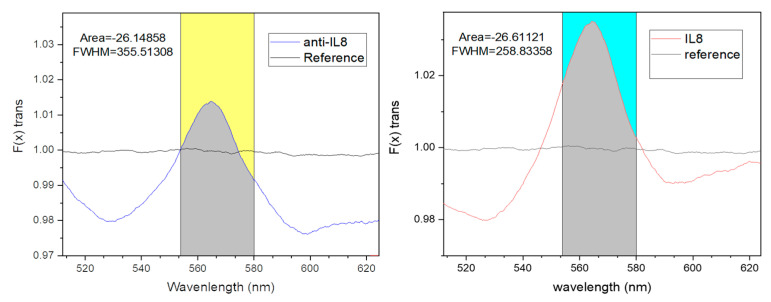
Left, Ftrans (per-unit) obtained after anti-IL-8 saturation on the surface. Right, Ftrans obtained after IL-8 recognition. The variation in the Ftrans area for the wavelength range (554 to 580 nm, gray area) for this case was 0.46263 nm (26.61121 nm–26.14858 nm).

Contrary to the approach utilized in our previous works, here, we applied a completely novel signal processing approach to the biosensing results, where the collected signal is analyzed and treated to improve the detection limit. This means that the readout signal is not derived from the position of the resonant mode’s wavelength but from the entire area under the Ftrans curve per unit. Ftrans represents the resonance mode curve produced by the RNPs at different biosensing stages, such as antibody immobilization and the recognition of varying analyte concentrations, divided by the resonant mode curve of the reference RNPs. The reference RNPs are RNPs measured without any biological element bonded to their surface and in contact with a buffer. This processing method is being reported here for the first time to improve the detection limit for the described continuous label-free biosensor (Figure 6). The average variation in the baseline or background is significantly less than the variation in a single point on this curve. In addition, we had to use a range of wavelengths in which the area of the Ftrans curve changed significantly depending on the biosensor response, providing better reliability and robustness for monitoring the biosensing signal. The area of the Ftrans resulted in a better figure of merit for improving the sensitivity compared to the previous methods reported [25].

### 3.3. Continuous IL-8 Biosensing Measurements

**Figure 7:**
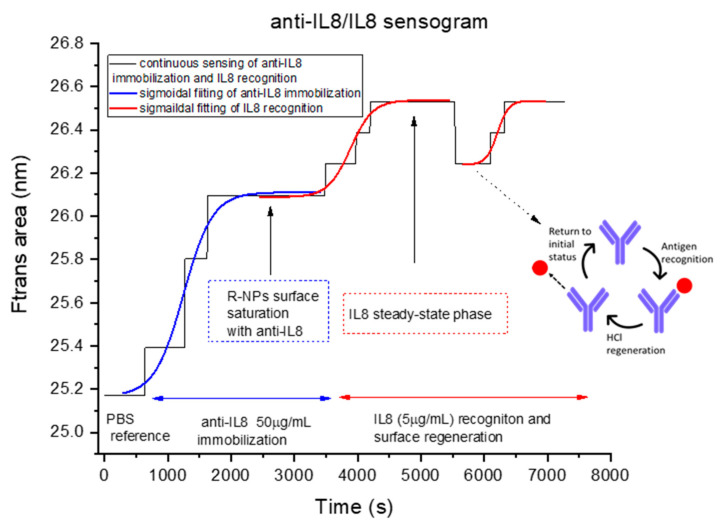
Continuous sensing of anti-IL-8 immobilization, subsequent IL-8 detection, and regeneration process as a function of time (sensogram) through resonant mode position continuous monitoring.

For the sensogram acquisition, we monitored the area of the Ftrans per unit previously defined, resulting from the optical interrogation of the RNPs, as a function of time (Figure 7). For the continuous biosensing experiment (based on molecule affinity), including the regeneration step, the order of the liquids flowed subsequently, and the starting flowing times (sec) were: PBS (sec 0), anti-IL-8 (50 µg/mL) (sec 500), IL-8 (5 µg/mL) (sec 3500), HCl (sec 5500), and IL-8 (sec 6200). After every biosensing step or flow of different reagents, PBS flowed through the system for 60 s to avoid cross-contamination between consecutive fluids.

Successful immobilization was verified by monitoring the change in the Ftrans signal after introducing the anti-IL-8 antibody into the fluidic device. A distinct shift in the Ftrans area was observed, confirming antibody attachment to the sensor surface. Subsequently, when IL-8 flowed through the device, an additional Ftrans change occurred, demonstrating that the immobilized antibody specifically recognized and bound to its target analyte. These consecutive optical responses validate both the immobilization process and the specificity of the IL-8 recognition.

Figure 7 shows the complete sensogram, including every biological step carried out. The Ftrans area has increased by 0.92 nm after anti-IL-8 antibody saturation. Then, when the IL-8 antigen was flowed and then bound to anti-IL8, the area of Ftrans increased again, 0.46 nm. This was the recognition step of the immunoassay. Lastly, an HCl solution was injected into the system to encourage the dissociation of anti-IL8/IL8 and repeat the detection of IL-8 through a new recognition phase of the anti-IL8.

**Figure 8:**
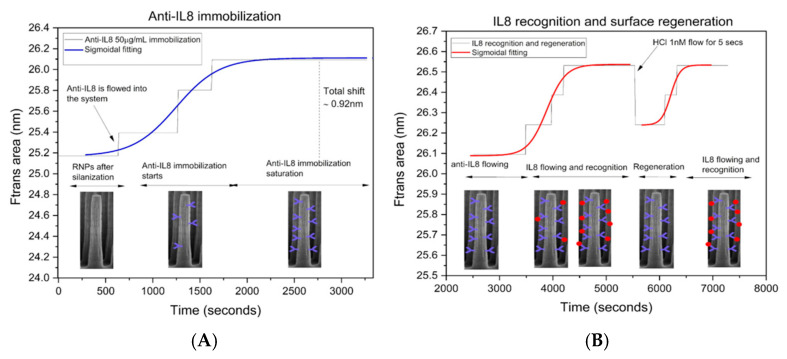
(**A**) Anti-IL8 progressive immobilization on RNP surface. (**B**) IL8 recognition and surface for repeated detection processes.

In Figure 8A, the anti-IL-8 immobilization process can be observed in detail. First, we flowed PBS as a reference signal of the sensogram for 500 s (around 8 min). Subsequently, we flowed anti-IL-8 at a flow rate of 50 µL/min for 2500 s (41 min). In the last 10 min of this step, the anti-IL-8 solution was replaced with BSA at a flow rate of 100 µL/min for surface-blocking purposes. The RNPs’ Ftrans area increased from 25.17 nm (in PBS) to 25.39 nm first, then to 25.8, and finally to 26.09 nm, which remained stable until the next liquid flow. This indicates that the surfaces of the RNPs were progressively functionalized with anti-IL-8 until the Ftrans area reached a plateau at 26.09 nm, signifying that the RNPs were completely covered with anti-IL-8 molecules during the incubation of the anti-IL-8 on the RNPs.

Figure 8B shows the association phase in detail. This phase includes the IL-8 recognition and the steady-state or equilibrium phase. IL-8 flowed into the system for 2000 s (33 min approx.). The Ftrans area increased from 26.09 nm to 26.24 and 26.38 as IL8 molecules were progressively recognized by the anti-IL8 before reaching a final area of 26.53 nm. After reaching the plateau, or so-called steady phase, 1 nM HCl was manually flowed with a syringe pump for five seconds to fill the chamber with the acidic solution. This would induce a pH change, facilitating the unbinding of biomolecules in the regeneration. This process does not affect the covalent bonds formed by the antibody and the inorganic surface of the RNPs, and thus, the sensing chip can be reused, and repeated sensing cycles can be performed. After the regeneration phase, the Ftrans area decreased to 26.24 nm, suggesting partial dissociation of the anti-IL-8 from the IL-8 analyte complex. The Ftrans area decreased to the value corresponding to antibody immobilization, 26.09 nm, for complete dissociation. The observed value indicates that some antibody–antigen complexes remained bound, likely due to inefficiencies in the regeneration process. Although this did not significantly affect the reliability of the system over multiple regeneration cycles, further optimization of the regeneration protocol may be explored in future studies to achieve full recovery to baseline. Various regeneration times were tested, and a 5 s duration was optimal, providing sufficient regeneration and allowing the system to proceed effectively into the recognition phase. A second recognition step after regeneration can be observed in Figure 8B from the second 5500 onward.

The **limit of detection (LoD)**, **specificity**, and **repeatability** are key performance indicators that determine the analytical reliability and practical applicability of any biosensor.

The LoD defines the lowest concentration of an analyte that can be reliably distinguished from the background noise, reflecting the sensitivity of the detection system. A low LoD is essential for detecting biomarkers present at trace levels in complex biological samples, particularly in early-stage disease diagnostics or real-time monitoring applications.

The system reached a limit of detection (LoD) of 22.7 ng/mL, which is within the levels of IL8 found in different cancer tissues, for example, in triple-negative breast cancer (TNBC) cultures [1]. LoD was calculated by considering the sensitivity of the IL8 detection and the uncertainty of the signal process methodology. The sensitivity (*m*) was calculated as the slope between the variation of the area of Ftrans of the RNPs when saturated with anti-IL8 and the area of the Ftrans after the IL-8 recognition ($\Delta_{AFtrans}$ and the variation of detected biomolecule concentration $\left(\Delta (IL8)\right).$

(1) $$m=\frac{\;\;\Delta_{AFtrans}\;}{\Delta \;\left[IL8\right]}=\frac{0.44\;nm}{5\;\mu g/mL}=0.088\;\left(\frac{nm}{\frac{\mu g}{mL}}\right)$$

The limit of detection (LoD) is the uncertainty of the measured signals (*U*) divided by the sensitivity.

(2) $$LoD=\frac{U}{m}=\frac{\;0.002\;(nm)}{0.088\;(\frac{nm}{\frac{\mu g}{mL}})}=0.022\;\frac{\mu g}{mL}=22.7\;ng/mL$$

In order to evaluate the uncertainty of the Ftrans area, it was necessary to estimate the Ftrans baseline variation for the spectral range defined (554 to 580 nm). Given that the system was working in continuous mode, we obtained a variation Ftrans baseline to determine the area of Ftrans variation along the experiment. It is worth mentioning here that Ftrans is determined by the ratio of the intensity of two interferometers (Isig/IRef), one used to monitor the signal (Isig) and the other one to monitor the reference signal (Iref), such the following equation [25]. For this paper, we employed the same RNP-based interferometer to obtain information at different times. This means acquiring the Isign signal multiple times, which is then mathematically normalized by the determined background Ftrans intensity variation (Δ intensity) and its corresponding Ftrans area variation. The output was a Ftrans baseline defined as around 1 on the X-axis, showing the system error on the Y-axis. Therefore, the Ftrans area uncertainty for the background for the spectral range (Δλ) was established.

The uncertainty of the Ftrans area was calculated as Δλ multiplied by Δ intensity. As mentioned above (Figure 6), Δλ is 554–580 nm = 26 nm as a constant, and the variation for Ftrans (Δ intensity), calculated as the peak-to-peak variation (1.00001–0.99982), resulted in 0.0001. Under these considerations, the area of Ftrans uncertainty (U) was estimated as 26 nm multiplied by 0.0001, resulting in an uncertainty, U, equal to 0.002. Figure 7 and Figure 8 show the detection of IL8 within minutes. IL8 is an effective biomarker for cancer prognosis estimation and therapy evaluation. This continuous platform for the detection of IL8 biomarkers represents a groundbreaking advancement with profound implications in the field of medical monitoring and beyond, which could be used for the growing organ-on-a-chip biomedical technology. The achievement of reaching a competitive limit of detection using a label-free optical biosensor signifies a monumental leap forward in the ability to monitor and understand inflammatory processes within the human body in a fast, cost-effective, and straightforward manner.

The specificity of a biosensor indicates its ability to selectively recognize the target analyte in the presence of structurally or functionally similar molecules. High specificity ensures that the sensor’s response arises solely from the intended biomolecular interaction (e.g., antigen–antibody binding), minimizing false-positive or cross-reactive signals that could compromise diagnostic accuracy.

The specificity of the biosensor shown here was ensured through the covalent immobilization of anti-IL-8 monoclonal antibodies and a BSA blocking step to suppress nonspecific binding. The distinct signal response between IL-8 and the control samples confirms the high specificity of the RNP network.

The repeatability (or intra-assay precision) assesses the consistency of the sensor’s response under the same experimental conditions. It reflects the robustness of the biosensing platform, ensuring reproducible performance across multiple cycles of detection and regeneration. High repeatability is crucial for the sensor’s translation into reliable, real-world applications.

The repeatability of the detection system was verified by performing a regeneration cycle using HCl injections. The sensor maintained consistent optical responses and reproducible Ftrans variations over successive measurements, demonstrating robust performance.

While this study utilized purified recombinant IL-8 to establish the foundational performance of the assay, future work will involve testing in complex biological matrices, such as serum, tumor tissue fluid, and organ–chip secretions, to evaluate potential matrix effects and nonspecific adsorption.

When applying the system to biological fluids such as serum, plasma, or tumor-conditioned medium, challenges such as nonspecific adsorption, biofouling, and matrix effects may interfere with the optical signal and reduce specificity. To address these limitations, our future work will focus on

- Surface passivation and blocking strategies, including the use of PEG-based coatings to minimize nonspecific binding;
- Regeneration and cleaning protocols, such as low-pH or detergent-based rinses, to avoid biofouling and preserve the sensor’s reusability;
- Optimization of sample preparation, which will include testing different dilution factors and various buffer compositions to identify the optimal conditions for diluting real samples while maintaining biomarker detectability;
- Generation of matrix-matched calibration curves using spiked biological samples to account for potential refractive index and background variations.

These studies will be critical for validating the continuous sensing platform’s applicability in real-world diagnostic and research scenarios.

## 4. Conclusions

The proposed biosensing module represents a transformative leap in the field of biosensing, leading to a new era of continuous, multiplexed, and highly sensitive monitoring. The biosensing module developed in this work represents a quantum leap in design and fabrication. The development of a completely new, fluidic-integrated, and portable device marks a paradigm shift in point-of-care testing and remote monitoring. Its compact and portable nature means that it can be effortlessly deployed in various settings, such as organ-on-a-chip fluidic platforms.

We developed microfluidic label-free biosensors that eliminate the need for exogenous labels, reducing biosensing times from several hours to minutes, the potential damage to biological samples, and the cost associated with the excessive use of reagents. Moreover, the capacity of the presented biosensing module to regenerate makes it invaluable in studies involving chronic diseases or the assessment of dynamic biological processes. It can provide an uninterrupted stream of data, revealing trends and patterns that might be missed with traditional endpoint measurements.

This work successfully validated the core functionalities of the biosensing platform described here and demonstrated its potential for continuous detection applications. Key achievements include the following:

- Validation of Fluidic and Optical Systems: The fluidic system was shown to operate reliably without leakage, with precise liquid flow control. Optical alignment and the functionality of the RNPs were confirmed through bulk and dynamic sensing experiments.
- Demonstration of Sensitivity: Bulk sensing experiments revealed a sensitivity of 350 nm/RIU, consistent with or exceeding comparable systems in the literature. This highlights the robustness of the sensor’s design and its effectiveness in refractive index detection.

Continuous Detection Stability: The biosensing module maintained stable operation O que bienover time, proving its capability for extended continuous measurements. The system also demonstrated reproducibility across various liquids (water, ethanol, and IPA), underscoring its reliability.

- Software Integration: The integration of custom MATLAB-based continuous measurement software enhanced the platform’s data acquisition and processing capabilities, enabling seamless operation and robust analysis. In addition, we implemented an innovative and unconventional data processing approach using Ftrans to significantly enhance the LoD of the IL-8, reaching 22.7 ng/mL. This pioneering method represents a substantial advancement in precisely quantifying IL-8 levels found in the microfluidic environment of organ-on-a-chip systems, and its performance aligns with other label-free biosensors for interleukin detection. The Table 1 below summarizes the limit of detection (LoD) for interleukins from other reported label-free biosensors.

IL-8 is a critical proinflammatory cytokine that is a crucial indicator of the body’s immune response. The ability to detect IL-8 biomarkers continuously, especially at a highly competitive limit of detection, offers unparalleled precision in diagnosing and managing a broad spectrum of diseases and conditions. This encompasses inflammatory disorders, infections, autoimmune diseases, and certain cancers.

The obtained limit of detection (LoD) of 22.7 ng/mL is competitive and falls within the physiologically relevant range of IL-8 concentrations reported in cancer-related biological fluids. Clinical studies have shown that IL-8 levels in serum and plasma typically range from 10 to 100 ng/mL in patients with advanced cancers, such as breast, lung, and colon carcinoma, whereas early-stage or low-grade tumors may exhibit IL-8 concentrations in the sub-ng/mL to low-ng/mL range. Therefore, the LoD achieved in this work makes the platform particularly suitable for continuous monitoring of IL-8 in models reproducing moderate-to-high cytokine expression levels, such as tumor-on-a-chip systems or therapeutic response assays. Nevertheless, for early diagnostic applications, where IL-8 may be present at trace concentrations, future efforts will focus on enhancing the platform’s sensitivity through optical coupling improvements, optimizing nanopillar geometry, and refining data-processing algorithms to further lower the detection threshold toward sub-ng/mL levels.

When compared to existing biosensing platforms, our system offers several distinct advantages. For instance, traditional label-based methods, such as ELISA, often require several hours for detection and involve complex reagent handling, whereas our label-free approach reduces detection times to minutes and simplifies the workflow. Furthermore, the integration of microfluidics and continuous monitoring capabilities sets our platform apart from conventional endpoint measurement systems, enabling real-time tracking of dynamic biological processes.

Compared with other label-free optical biosensing technologies, including optical fiber, photonic crystal, and interferometric systems [29,30,31], the RNP-based platform developed in this work offers notable advantages for continuous cancer biomarker detection. While recent approaches, such as dielectric grating sensors and bimodal interferometric photonic sensors, provide high sensitivity [32], they typically require complex optical coupling and are not easily integrated into microfluidic or organ-on-a-chip devices. In contrast, the RNP platform employs vertical light interrogation within a compact and modular architecture, allowing for straightforward integration and true continuous real-time operation. Additionally, the novel Ftrans-based signal processing improves detection robustness and sensitivity compared to conventional wavelength-shift analysis. Together, these features position the RNP biosensor as a scalable and integration-ready alternative for the continuous monitoring of cancer-related biomarkers.

However, certain limitations should be acknowledged. For example, while the platform demonstrates excellent performance in controlled environments, its application in complex biological matrices (e.g., serum or plasma) may require further optimization to mitigate potential interference from nonspecific binding or matrix effects. Future work will focus on addressing these challenges through surface functionalization strategies and advanced data-processing algorithms.

The platform’s high sensitivity, reproducibility, and adaptability make it a promising tool for cancer monitoring in clinical settings. For example, it could be integrated into routine diagnostic workflows to measure biomarker levels in patient samples, such as serum or tumor tissue fluid, enabling real-time monitoring of disease progression and treatment responses.

The system’s modular design allows for easy adaptation to different clinical layouts and facilities, as well as for different biomarkers, making it suitable for personalized medicine approaches. Simultaneous monitoring of a high number of biomarkers in a single biosensing system is a challenge due to the integration of multiple biosensors and the complexity of the optical readout implementation. Its potential for multiplexed detection could facilitate comprehensive cancer profiling, providing clinicians with a more holistic view of the disease, as well as benefits to help diagnose, monitor progression, determine risk factors, and assess prognosis. To support clinical adoption, future work will focus on validating the platform in large-scale clinical studies, optimizing sample preparation protocols, and ensuring compliance with regulatory standards.

The integration of the proposed continuous biosensing platform into cancer-on-a-chip systems represents a significant step toward real-time and physiologically relevant monitoring of tumor microenvironments. Continuous IL-8 detection offers dynamic information that traditional endpoint assays, such as ELISA, cannot provide, allowing for the observation of cytokine secretion patterns over time under varying biological or therapeutic conditions. Such integration would enable the study of tumor–stroma and tumor–immune interactions, as well as the inflammatory responses induced by anticancer or immunomodulatory drugs within microphysiological systems. By coupling the biosensing module directly to the effluent streams of organ-on-a-chip devices, non-invasive, label-free tracking of cytokine levels could be achieved, providing a more accurate and temporally resolved understanding of tumor behavior and therapeutic efficacy. Future work will focus on implementing this approach in established cancer-on-a-chip models to correlate IL-8 secretion dynamics with treatment response, thereby enhancing the predictive power of these in vitro platforms for drug screening and personalized medicine applications.

In summary, the continuous detection of IL-8 biomarkers at a competitive limit of detection is a monumental achievement that transcends the boundaries of traditional diagnostics. Its potential impact on healthcare is immeasurable, revolutionizing disease management and early intervention strategies. The novel design of a portable and integrative fluidic device creates a new era in healthcare technology, where precision and connectivity converge to empower healthcare providers and improve patient outcomes on an unprecedented scale.

## Figures and Tables

**Table 1:** LoDs of other label-free biosensors for IL-8 detection.

	LoD
Multimodal Platform	22.7 ng/mL
SPR ProteOn™ XPR36 Protein Interaction Array System [26]	8.4–84 ng/mL
Interferometric Reflectance Imaging Sensor (IRIS) [27]	19 ng/mL
Electrochemical Detection [28]	72.73 pg/mL

## Data Availability

The original contributions presented in this study are included in the article. Further inquiries can be directed to the corresponding authors.

## References

[1] Cheng Y., Ma X.L., Wei Y.Q., Wei X.W. (2019). Potential roles and targeted therapy of the CXCLs/CXCR2 axis in cancer and inflammatory diseases. Biochim. Biophys. Acta (BBA)-Rev. Cancer.

[2] Matsushima K., Yang D., Oppenheim J.J. (2022). Interleukin-8: An evolving chemokine. Cytokine.

[3] Todorović-Raković N., Milovanović J. (2013). Interleukin-8 in breast cancer progression. J. Interferon Cytokine Res..

[4] Sahibzada H.A., Khurshid Z., Khan R.S., Naseem M., Siddique K.M., Mali M., Zafar M.S. (2017). Salivary IL-8, IL-6 and TNF-α as Potential Diagnostic Biomarkers for Oral Cancer. Diagnostics.

[5] Kawakami M., Suzuki N., Sudo Y., Shishido T., Maeda M. (1998). Development of an enzyme-linked immunosorbent assay (ELISA) for antitumor agent MKT 077. Anal. Chim. Acta.

[6] Hosseini S., Vázquez-Villegas P., Rito-Palomares M., Martinez-Chapa S.O. (2018). Advantages, disadvantages and modifications of conventional ELISA. Enzyme-Linked Immunosorbent Assay (ELISA) from A to Z.

[7] Kaur J., Preethi M., Srivastava R., Borse V. (2022). Role of IL-6 and IL-8 biomarkers for optical and electrochemical based point-of-care detection of oral cancer. Biosens. Bioelectron. X.

[8] Sang S., Wang Y., Feng Q., Wei Y., Ji J., Zhang W. (2015). Progress of new label-free techniques for biosensors: A review. Crit. Rev. Biotechnol..

[9] Rusling J.F., Kumar C.V., Gutkind J.S., Patel V. (2010). Measurement of biomarker proteins for point-of-care early detection and monitoring of cancer. Analyst.

[10] Naresh V., Lee N. (2021). A Review on Biosensors and Recent Development of Nanostructured Materials-Enabled Biosensors. Sensors.

[11] Rui M., Zhengyong L., Luis P., Chenkun Y., Sui Q., Carlos M. (2021). Optical fiber sensing for marine environment and marine structural health monitoring: A review. Opt. Laser Technol..

[12] Qu J.H., Ordutowski H., Van Tricht C., Verbruggen R., Barcenas Gallardo A., Bulcaen M., Ciwinska M., Gutierrez Cisneros C., Devriese C., Guluzade S. (2022). Point-of-care therapeutic drug monitoring of adalimumab by integrating a FO-SPR biosensor in a self-powered microfluidic cartridge. Biosens. Bioelectron..

[13] Wang L.J., Chang Y.C., Sun R., Li L. (2017). A multichannel smartphone optical biosensor for high-throughput point-of-care diagnostics. Biosens. Bioelectron..

[14] Mahzabeen F., Vermesh O., Levi J., Tan M., Alam I.S., Chan C.T., Gambhir S.S., Harris J.S. (2021). Real-time point-of-care total protein measurement with a miniaturized optoelectronic biosensor and fast fluorescence-based assay. Biosens. Bioelectron..

[15] Idili A., Montón H., Medina-Sánchez M., Ibarlucea B., Cuniberti G., Schmidt O.G., Plaxco K.W., Parolo C. (2022). Continuous monitoring of molecular biomarkers in microfluidic devices. Prog. Mol. Biol. Transl. Sci..

[16] Xu S., Zhan J., Man B., Jiang S., Yue W., Gao S., Guo C., Liu H., Li Z., Wang J. (2017). Real-time reliable determination of binding kinetics of DNA hybridization using a multi-channel graphene biosensor. Nat. Commun..

[17] Lubken R.M., de Jong A.M., Prins M.W. (2020). Multiplexed continuous biosensing by single-molecule encoded nanoswitches. Nano Lett..

[18] Zhang Y.S., Aleman J., Shin S.R., Kilic T., Kim D., Mousavi Shaegh S.A., Massa S., Riahi R., Chae S., Hu N. (2017). Multisensor-integrated organs-on-chips platform for automated and continual in situ monitoring of organoid behaviors. Proc. Natl. Acad. Sci. USA.

[19] Armstrong R.E., Horáček M., Zijlstra P. (2020). Plasmonic Assemblies for Real-Time Single-Molecule Biosensing. Small.

[20] Hernandez A.L., Dortu F., Veenstra T., Ciaurriz P., Casquel R., Cornago I., Horsten H.V., Tellechea E., Maigler M.V., Fernández F. (2019). Automated chemical sensing unit integration for parallel optical interrogation. Sensors.

[21] Hernández A.L., Casquel R., Holgado M., Cornago I., Sanza F.J., Santamaría B., Maigler M., Fernández F., Lavín A., Laguna M.F. (2015). Arrays of resonant nanopillars for biochemical sensing. Opt. Lett..

[22] Subia B., Dahiya U.R., Mishra S., Ayache J., Casquillas G.V., Caballero D., Reis R.L., Kundu S.C. (2021). Breast tumor-on-chip models: From disease modeling to personalized drug screening. J. Control. Release.

[23] Xing Y., Zhao L., Cheng Z., Lv C., Yu F., Yu F. (2021). Microfluidics-based sensing of biospecies. ACS Appl. Bio Mater..

[24] Hernandez A.L., Pujari S.P., Laguna M.F., Santamaría B., Zuilhof H., Holgado M. (2021). Efficient chemical surface modification protocol on SiO2 transducers applied to MMP9 biosensing. Sensors.

[25] Holgado M., Sanza F.J., López A., Lavín A., Casquel R., Laguna M.F. (2014). Description of an advantageous optical label-free biosensing interferometric read-out method to measure biological species. Sensors.

[26] ProteOn™ XPR36 Protein Interaction Array System—Bio-Rad. https://www.bio-rad.com/es-es/product/proteon-xpr36-protein-interaction-array-system?ID=ea380548-08ca-4b4e-896b-87e5580ac411.

[27] Ahn S., Freedman D.S., Massari P., Cabodi M., Ünlü M.S. (2013). A Mass-Tagging Approach for Enhanced Sensitivity of Dynamic Cytokine Detection Using a Label-Free Biosensor. Langmuir.

[28] Verma S., Singh A., Shukla A., Kaswan J., Arora K., Ramirez-Vick J., Singh P., Singh S.P. (2017). Anti-IL8/AuNPs-rGO/ITO as an immunosensing platform for noninvasive electrochemical detection of oral cancer. ACS Appl. Mater. Interfaces.

[29] Lyu S., Wu Z., Shi X., Wu Q. (2022). Optical Fiber Biosensors for Protein Detection: A Review. Photonics.

[30] Rafiee E. (2024). Photonic Crystal based Biosensor for Diagnosis of Kidney Failure and Diabetes. Plasmonics.

[31] Liu B., Li Y., Wang R., Chen X., Li J., Chen H., Jiang M. (2024). Label-free and selective heparin detection by surface functionalized fiber Fabry-Perot interferometer biosensor. Opt. Fiber Technol..

[32] di Toma A., Brunetti G., Colapietro P., Ciminelli C. (2024). High-resolved near-field sensing by means of dielectric grating with a box-like resonance shape. IEEE Sens. J..

